# Drug Susceptibility in Leishmania Isolates Following Miltefosine Treatment in Cases of Visceral Leishmaniasis and Post Kala-Azar Dermal Leishmaniasis

**DOI:** 10.1371/journal.pntd.0001657

**Published:** 2012-05-22

**Authors:** Vasundhra Bhandari, Arpita Kulshrestha, Deepak Kumar Deep, Olivia Stark, Vijay Kumar Prajapati, V. Ramesh, Shyam Sundar, Gabriele Schonian, Jean Claude Dujardin, Poonam Salotra

**Affiliations:** 1 National Institute of Pathology, Indian Council of Medical Research, Safdarjung Hospital Campus, New Delhi, India; 2 Institute of Microbiology and Hygiene, Charite University Medicine Berlin, Berlin, Germany; 3 Institute of Medical Sciences, Banaras Hindu University, Varanasi, India; 4 Department of Dermatology, Safdarjung Hospital, New Delhi, India; 5 Unit of Molecular Parasitology, Department of Parasitology, Institute of Tropical Medicine, Antwerp, Belgium; University of Pittsburgh, United States of America

## Abstract

**Background:**

With widespread resistance to antimonials in Visceral Leishmaniasis (VL) in the Indian subcontinent, Miltefosine (MIL) has been introduced as the first line therapy. Surveillance of MIL susceptibility in natural populations of *Leishmania donovani* is vital to preserve it and support the VL elimination program.

**Methodology and Principal Findings:**

We measured *in vitro* susceptibility towards MIL and paromomycin (PMM) in *L. donovani* isolated from VL and PKDL, pre- and post-treatment cases, using an amastigote-macrophage model. MIL susceptibility of post-treatment isolates from cured VL cases (n = 13, mean IC_50_±SD = 2.43±1.44 µM), was comparable (p>0.05) whereas that from relapses (n = 3, mean IC_50_ = 4.72±1.99 µM) was significantly higher (p = 0.04) to that of the pre-treatment group (n = 6, mean IC_50_ = 1.86±0.75 µM). In PKDL, post-treatment isolates (n = 3, mean IC_50_ = 16.13±2.64 µM) exhibited significantly lower susceptibility (p = 0.03) than pre-treatment isolates (n = 5, mean IC_50_ = 8.63±0.94 µM). Overall, PKDL isolates (n = 8, mean IC_50_ = 11.45±4.19 µM) exhibited significantly higher tolerance (p<0.0001) to MIL than VL isolates (n = 22, mean IC_50_ = 2.58±1.58 µM). Point mutations in the miltefosine transporter (LdMT) and its beta subunit (LdRos3) genes previously reported in parasites with experimentally induced MIL resistance were not present in the clinical isolates. Further, the mRNA expression profile of these genes was comparable in the pre- and post-treatment isolates. Parasite isolates from VL and PKDL cases were uniformly susceptible to PMM with respective mean IC_50_ = 7.05±2.24 µM and 6.18±1.51 µM.

**Conclusion:**

The *in vitro* susceptibility of VL isolates remained unchanged at the end of MIL treatment; however, isolates from relapsed VL and PKDL cases had lower susceptibility than the pre-treatment isolates. PKDL isolates were more tolerant towards MIL in comparison with VL isolates. All parasite isolates were uniformly susceptible to PMM. Mutations in the LdMT and LdRos3 genes as well as changes in the expression of these genes previously correlated with experimental resistance to MIL could not be verified for the field isolates.

## Author Summary

Resistance to antimonials has emerged as a major hurdle to the treatment and control of VL and led to the introduction of Miltefosine as first line treatment in the Indian subcontinent. MIL is an oral drug with a long half-life, and it is feared that resistance may emerge rapidly, threatening control efforts under the VL elimination program. There is an urgent need for monitoring treatment efficacy and emergence of drug resistance in the field. In a set of VL/PKDL cases recruited for MIL treatment, we observed comparable drug susceptibility in pre- and post-treatment isolates from cured VL patients while MIL susceptibility was significantly reduced in isolates from VL relapse and PKDL cases. The PKDL isolates showed higher tolerance to MIL as compared to VL isolates. Both VL and PKDL isolates were uniformly susceptible to PMM. MIL transporter genes LdMT/LdRos3 were previously reported as potential resistance markers in strains in which MIL resistance was experimentally induced. The point mutations and the down-regulated expression of these transporters observed *in vitro* could, however, not be verified in natural populations of parasites. LdMT/LdRos3 genes therefore, do not appear to be suitable markers so far for monitoring drug susceptibility in clinical leishmanial isolates.

## Introduction

Visceral Leishmaniasis (VL) is a potentially fatal protozoan infection with members of the *Leishmania donovani* complex as the causative species. This poverty related disease is endemic in 70 countries with a total of 200 million people at risk and an estimated 100,000 new infections annually concerning all age groups [1], [2]. More than 90% of the estimated VL cases occur in India, Bangladesh, Nepal, Sudan and Brazil [3] with India alone sharing almost 50% of the world's total disease burden. Post-kala-azar dermal leishmaniasis (PKDL) is a dermal sequel of VL that develops in 5–15% of the cured VL patients in India and in 60% cured VL patients in Sudan and is considered to constitute a major parasite reservoir in these regions. In the present situation, chemotherapy is the key strategy for VL control due to the absence of vaccines and the limited impact of vector control [4]–[6]. The situation is particularly grave in Bihar, India, where more than 60% of VL patients do not respond to traditional first line antimonial therapy. The use of Amphotericin B and its liposomal formulations, although highly effective even in antimony unresponsive patients, has limitations because of its renal toxicity, high costs and inconvenience due to slow I.V. based administration [7], [8].

The first oral antileishmanial drug Miltefosine (MIL), an alkylphosphocholine, has proved to be highly effective against VL with cure rates of 94%, including cases unresponsive to antimony [2], [9]. It was therefore, proposed as the first line VL therapy and remains the mainstay in the Kala-azar elimination program, that aims to reduce the incidence of VL to 0.0001% in the endemic areas of the Indian subcontinent by the year 2015 [10], [11]. Efficacy of MIL has also been established for the treatment of PKDL in India [12], [13]. In absence of directly observed therapy (DOT), widespread misuse of this self-administered drug could contribute to the rapid emergence of MIL resistance in the field. Moreover, phase IV trial of MIL in India suggested doubling of the relapse and failure rate compared to phase III trials [8], [14]. Treatment failures (almost all relapses) were recently also observed in Nepal [15]. Alarmingly, VL and PKDL treatment failure and relapse cases have already surfaced in a significant fraction of MIL treated patients in India (unpublished data). Unresponsiveness to MIL has been reported for cutaneous cases due to *L. braziliensis* and *Leishmania (Viannia) guyanensis* in South America [16], [17]. Paromomycin (PMM) is an aminoglycoside antibiotic exhibiting high efficacy towards VL [18]. Data from Phase IV trials confirm the safety and efficacy of PMM to treat VL [19].

The development and spread of drug resistance has made surveillance of drug susceptibility a high priority. In the present investigation, through long-term monitoring of MIL treated VL/PKDL patients, post-treatment stage parasite isolates were obtained either as residual parasites soon after completion of treatment or from cases that relapsed, and were compared to a set of pre-treatment isolates. The MIL susceptibility of these isolates was assessed with an *in vitro* intracellular amastigote assay and correlated with the clinical outcome. In addition, intrinsic sensitivity towards PMM was evaluated in the same set of clinical isolates in order to obtain baseline susceptibility prior to its future use in therapy.

An impaired functioning of P-Type ATPase transporters, the LdMT–LdRos3-dependent flippase machinery, resulting in a significant decrease of intracellular MIL concentration was observed in experimentally induced MIL resistant leishmanial parasites. [20], [21]. The resistant phenotype was related to the occurrence of two missense and a nonsense point mutations in the LdMT gene, and a nonsense mutation in the start codon of the LdRos3 gene. In our study, we have evaluated the presence of these mutations and the mRNA expression levels of LdMT and LdRos3 in our set of clinical isolates to explore their role as molecular markers for monitoring MIL susceptibility in the field.

## Methods

### Patients and parasites

Clinical isolates of *L. donovani* were prepared from splenic aspirates of VL patients reporting to KAMRC, Muzzafarpur, Bihar or from dermal lesions of PKDL patients reporting to Department of Dermatology, Safdarjung Hospital (SJH), New Delhi under the guidelines of the Ethical Committee of the respective Institute. All patients came from zones of high endemicity in Bihar, India. VL patients received MIL treatment for 28 days (50 mg capsule twice) while PKDL patients received MIL for 60 days (50 mg capsule, thrice daily). Splenic smears from all VL patients were examined microscopically at the pre and post-treatment stages. Patients with negative or +1 smear grade were not treated further. However, patients with smears grading ≥2 [22] were treated with amphotericin B deoxycholate.

Parasites isolated before onset of treatment were assigned XXX/0 codes. MIL treatment led to complete subsidence of VL symptoms, interpreted as clinical cure, although residual parasites could be cultured from splenic aspirates in a substantial number of patients at the end of 1 month of treatment. These post-treatment isolates were assigned with XXX/1 codes. All cases were followed up for one year. VL and PKDL cases that relapsed after an initial cure were treated with Amphotericin B and cured. Parasites were isolated from each of the relapse cases at the time of reported relapse (after four, six and seven months of MIL treatment completion in VL and after 12, 18 and 32 months of MIL treatment in PKDL and were designated as XXX/month in which relapse occurred.

Parasites were routinely grown in Medium 199 (Sigma) with 10% heat-inactivated fetal bovine serum (HI FBS, Gibco, USA) at 25°C.

### Species characterization

Parasite DNA, isolated using Qiagen Kit, was subjected to ITS-1 PCR-RFLP analysis for species characterization, as described earlier [23]. The ITS1 PCR product was digested with 1 U *Hae*III enzyme (Genei, Bangalore, India) at 37°C for 2 hours, followed by analysis on 2% agarose gel. All isolates were characterized as *L. donovani* on the basis of their RFLP pattern.

### Drug susceptibility assay at intracellular amastigote stage

The drug susceptibility of *L. donovani* parasites was assessed as intracellular amastigotes, as previously described [24]. Briefly, J774A.1 cells (1×10^5^ cells/ml) were infected with stationary phase promastigotes at ratio of 10 ∶ 1 (parasite ∶ macrophage), plated into 16 well chambered Labtek tissue culture slides and incubated for 4 h at 37°C in 5% CO_2_. Excess, non-adhered promastigotes were removed by washing and macrophages incubated for 18–24 h. Infected cells were re-incubated for 48 h, with MIL (1, 5, 10, 20 and 30 µM) (Paladin) or PMM (1, 5, 10, 20, 30 and 40 µM) (Gland Pharma). Macrophages were then examined for intracellular amastigotes after staining with Diff-Quik solutions. The number of *L. donovani* amastigotes was counted in 100 macrophages, at 100× magnification. The survival rate of parasites relative to untreated macrophages was calculated and IC_50_ were determined by sigmoid regression analysis. The assays were performed in duplicate and repeated at least twice.

### Partial sequencing of LdMT and LdRos3 genes

Fourteen parasite isolates were tested for the presence of previously described point mutations in the LdMT gene, at positions T421N, L856P and W210*, and the LdRos3 gene, at position M1*(Figure S1) [20], [21]. Primers were designed targeting these mutations and their adjacent regions using Primer 3 software [25]. PCR reactions were performed with 35 ng of genomic DNA in a 50 µl reaction volume containing 10× PCR buffer (incl. 15 mM MgCl_2_), 5 U/µl of *Taq* polymerase (Roche), 2.5 mM dNTPs (NEB), and 10 µM of each of the locus specific primers (Table S1). Thermocycling conditions were as follows: initial denaturation at 95°C for 5 min, followed by 35 cycles of denaturation at 95°C for 30 sec, annealing with the specific primer pair at the specific T_A_°C for 30 sec and extension at 72°C for 1 min, and a final extension step at 72°C for 6 min. PCR products with fragment sizes between 149 and 277 bp were purified (QIAamp DNA Mini Kit, Qiagen) and sent for commercial sequencing using forward and reverse primers (SMB Services in Molecular Biology, Berlin Germany). Sequences were checked, trimmed and aligned using Chromas Pro v 1.32 [26].

### RNA extraction and real-time PCR analysis

Total RNA (5 µg), isolated from stationary phase promastigotes at day six using Trizol Reagent (Invitrogen, USA) was reverse transcribed at 42°C with M-MLV Reverse transcriptase (Invitrogen, USA) after deoxyribonuclease I treatment. All real-time PCR reactions were performed in duplicate in 25 µl volumes using SYBR Green as described before [27]. The 2^−ΔΔCT^ method was used to calculate relative changes in gene expression determined from real-time quantitative PCR experiments. The data was presented as the fold change in the target gene expression in *L.donovani* parasites normalized to the internal control gene (GAPDH) and relative to the *Ld*AG83 reference strain of *L. donovani*.

### Ethical statement

The study was approved by the Ethical Committee of the Institute of Medical Sciences, Banaras Hindu University, Varanasi and Safdarjung Hospital, New Delhi, India. Written informed consent was obtained from patients and from guardians in case of children <18 years.

## Results

### *In vitro* susceptibility to MIL

MIL susceptibility was determined at intracellular amastigote stage for thirty clinical isolates, including eleven pre-treatment isolates (six from VL and five from PKDL cases), thirteen post-treatment isolates (obtained at the end of treatment from VL patients which depicted a clinical cure, although parasitology was still positive) and six relapse isolates (three VL and three PKDL). The clinical profile of patients and *in vitro* susceptibility of the isolates to MIL are summarized in Table 1.

**Table 1:** Drug susceptibility of *Leishmania donovani* clinical isolates following Miltefosine treatment in cases of Visceral Leishmaniasis and Post kala-azar dermal Leishmaniasis.

WHO code^1^	Research code^2^	Patient age/sex	Clinical response to treatment	MIL	PMM
				IC_50_±SD (µM)	IC50±SD (µM)
MHOM/IN/2010/BHU782/0	V782/0	18/M	Cure	0.95±0.06	5.34+1.12
MHOM/IN/2010/BHU869/0	V869/0	10/F	Cure	1.05±0.08	8.97±0.25
MHOM/IN/2010/BHU994/0	V994/0	60/M	Cure	1.96±0.50	9.07±0.15
MHOM/IN/2010/BHU828/0	V828/0	10/M	Cure	1.98±0.66	5.06±0.37
MHOM/IN/2010/BHU902/0	V902/0	7/M	Cure	2.30±0.36	10.70±1.12
MHOM/IN/2009/BHU815/0	V815/0	12/F	Cure	2.91±0.24	7.25±0.55
MHOM/IN/2010/BHU796/1	V796/1	NA	Cure	1.02±0.17	5.55±0.89
MHOM/IN/2010/BHU1121/1	V1121/1	12/M	Cure	1.14±0.11	6.05±0.65
MHOM/IN/2010/BHU807/1	V807/1	12/F	Cure	1.26±0.15	9.08±0.65
MHOM/IN/2010/BHU869/1	V869/1	10/F	Cure	1.32±0.26	3.91±0.03
MHOM/IN/2010/BHU1042/1	V1042/1	6/M	Cure	1.42±0.06	4.23±0.76
MHOM/IN/2009/BHU815/1	V815/1	12/F	Cure	2.27±0.49	5.88±0.64
MHOM/IN/2009/BHU741/1	V741/1	NA	Cure	2.35±0.40	6.07±1.02
MHOM/IN/2009/BHU800/1	V800/1	35/M	Cure	2.48±0.04	9.80±0.61
MHOM/IN/2010/BHU902/1	V902/1	7/M	Cure	3.37±0.38	9.28±0.40
MHOM/IN/2010/BHU1093/1	V1093/1	20/M	Cure	3.57±0.35	3.41±0.29
MHOM/IN/2009/BHU994/1	V994/1	60/M	Cure	3.72±0.52	5.10±0.65
MHOM/IN/2010/BHU1080/1	V1080/1	12/M	Cure	4.95±0.28	5.80±0.84
MHOM/IN/2010/BHU814/1	V814/1	NA	Cure	5.20±0.80	9.20±0.75
MHOM/IN/2010/BHU1113/7	V1113/7	35/M	Relapse	2.67±0.51	7.02±0.86
MHOM/IN/2010/BHU872/6	V872/6	18/M	Relapse	4.84±0.39	10.70±1.02
MHOM/IN/2009/BHU1062/4	V1062/4	7/F	Relapse	6.66±0.62	7.64+0.76
MHOM/IN/1998/NIPP44/0	P44/0	18/M	Cure*	7.37±0.07	5.01±0.38
MHOM/IN/2011/NIPP232/0	P232/0	42/M	Cure	7.99±0.15	8.46±0.77
MHOM/IN/1998/NIPP48/0	P48/0	15/M	Cure*	8.88±0.18	5.80±0.85
MHOM/IN/1998/NIPP49/0	P49/0	23/M	Cure*	9.23±0.22	5.10±0.37
MHOM/IN/2001/NIPP93/0	P93/0	25/M	Cure*	9.69±0.52	6.21±0.37
MHOM/IN/2010/NIPP195/12	P195/12	21/M	Relapse	13.26±0.89	8.62±1.82
MHOM/IN/2010/NIPP214/18	P214/18	35/M	Relapse	16.70±0.65	4.92±0.34
MHOM/IN/2011/NIPP214/32	P214/32	36/M	Relapse	18.45±0.79	5.34±0.75

1 WHO code: country and year of isolation and the respective strain code, the number following the isolate ID indicates the number of months elapsed after start of MIL treatment.

2 Research code: Parasites cultured from VL patients were labeled V- and from PKDL patients P-, respectively. The number following the isolate ID indicates the number of months elapsed after start of MIL treatment (e.g. V902/1 means one month passed from first MIL treatment). Parasites isolated from patients' prior start of MIL treatment were labeled as XXX/0 and one month following first treatment was labeled XXX/1. These patients cleared from VL symptoms after respective duration of MIL treatment and were interpreted as clinical cure, although residual parasites could be cultured from splenic aspirates (marked XXX/1). In the period of 1 year follow up, cases of relapse were observed in three VL patients that had shown an initial clinical cure, the isolates obtained were designated as XXX/month in which relapse occurred.

Cure*- Patients treated with SAG (1000 mg intra muscularly), daily for four months.

**Figure 1:**
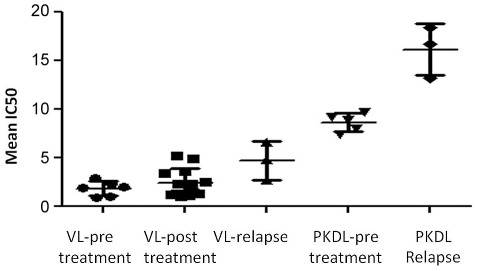
*In vitro* miltefosine susceptibility of parasite isolates from VL and PKDL cases before and after Mil treatment. Sensitivity of VL and PKDL isolates at intracellular amastigote stage were determined by infection in murine macrophage cell line J774A.1. Each individual value represents mean IC_50_±SD of the results from two separate assays.

The six VL pre-treatment isolates showed a sensitivity range of 0.95±0.06 to 2.91±0.24 µM towards MIL with the mean IC_50_±SD being 1.86±0.75 µM (Figure 1). The post-treatment VL isolates had a mean IC_50_ of 2.43±1.44 µM (range 1.02±0.17 to 5.20±0.80 µM) which was not significantly different in comparison with the pre-treatment group (p>0.05). The mean IC_50_ of the three VL isolates from relapse cases (4.72±1.99 µM) was significantly higher (p = 0.04) than that of pre-treatment VL cases (1.86±0.75 µM).

The three PKDL relapse isolates showed a significantly higher (p = 0.03), mean IC_50_ of 16.13±2.64 µM in comparison with the pre-treatment PKDL isolates (mean IC_50_ = 8.63±0.94 µM). The mean susceptibility of all VL isolates (2.58±1.58 µM, n = 22) when compared with PKDL isolates (11.45±4.19 µM, n = 8) revealed that the latter were significantly (p<0.0001) more tolerant to MIL (Figure 1).

### Natural susceptibility of *L. donovani* isolates to PMM

**Figure 2:**
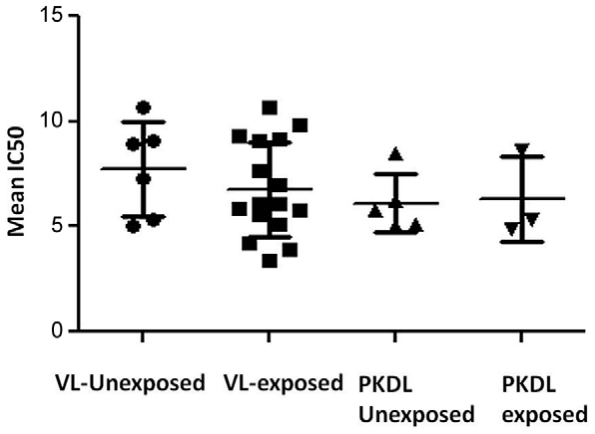
PMM susceptibility profile of VL and PKDL isolates exposed or non-exposed to MIL treatment. Susceptibility of VL and PKDL isolates at intracellular amastigote stage was determined by infection in murine macrophage cell line J774A.1. Each individual value represents mean IC_50_±SD of the results from two separate assays.

We evaluated PMM susceptibility of 22 VL isolates, of which 16 were exposed to MIL treatment. The IC_50_ ranged from 3.41±0.29 to 10.70±1.12 µM with mean IC_50_ = 7.05±2.24 µM (Table 1). Furthermore, the PMM sensitivity was similar (p>0.05) in parasites non-exposed (mean IC_50_ = 7.73±2.25 µM) or exposed (mean IC_50_ = 6.79±2.25 µM) to MIL (Figure 2).

The inherent PMM susceptibility of 8 PKDL isolates ranged from 4.92±0.34 to 8.62±1.82 µM (Table 1). Like in VL, the PMM IC_50_ of PKDL isolates was similar (p>0.05) in parasites non-exposed (n = 5, mean IC_50_ = 6.12±1.40 µM) or exposed (n = 3, mean IC_50_ = 6.29±2.02 µM) to MIL (Figure 2). There was no correlation observed between MIL and PMM susceptibility in VL (r = 0.10) or PKDL isolates (r = −0.02).

### Point mutations in the LdMT and LdRos3 genes possibly related to MIL resistance

None of the four reported SNPs in the LdMT and LdRos3 genes, which were suggested to be responsible for the resistant phenotype in a strain from Ethiopia, MHOM/ET/1967/HU3_MIL-R, with experimentally induced MIL resistance [21], could be detected in the clinical isolates investigated (Figure S1). Furthermore, no SNPs were detected when the three LdMT and the LdRos3 gene fragments were sequenced in 15 clinical isolates from Nepalese VL cases that relapsed after MIL treatment (data not shown). Notably, the LdRos3 gene fragment could not be amplified for the strains BHU800/1 and BHU1062/4 although no sequence polymorphisms were identified in the primer annealing sites.

### Expression of markers of experimental MIL resistance in field isolates

**Figure 3:**
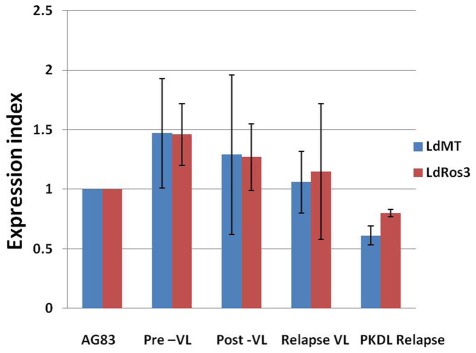
Expression of LdMT and LdRos3 in clinical isolates of VL and PKDL. Real-time reverse-transcription PCR expression analysis of *L. donovani* MIL transporter genes (LdMT and LdRos3) was performed using GAPDH as internal control. Graph shows the expression index, defined as ratio of gene expression relative to that of strain *Ld*AG83. Data represent the mean±SD of the results of three independent experiments.

mRNA expression level of LdMT and LdRos3 was analyzed in 19 VL and two PKDL isolates using real-time PCR in comparison to the reference strain *L. donovani Ld*AG83. The expression was found comparable in all the groups including the relapse cases of VL and PKDL (Figure 3).

## Discussion

The introduction of MIL therapy as treatment for VL has pioneered the era of effective oral therapy for this potentially fatal disease. However, anthroponotic VL transmission in the Indian subcontinent and the long half life of MIL (150–200 h) poses the risk of development of resistance in natural population of parasites. It has been reported earlier that MIL resistant parasites can be easily generated *in vitro* [28]. The present study reveals for the first time, the intrinsic *in vitro* sensitivity of Indian *L. donovani* isolates from a set of VL and PKDL patients treated with MIL (including both responders and relapse cases). The data provides information on the extent of MIL-tolerance in natural populations following MIL treatment highlighting the need for adequate monitoring of drug susceptibility to preserve this valuable drug.

The drug susceptibility of the currently prevailing clinical isolates was similar to the *L. donovani* parasites from the era of pre- MIL treatment reported earlier [27]. At the end of MIL treatment all cases showed clinical cure although some of them were parasitologically positive and residual parasites could be cultured from splenic aspirates of such patients (Table 1). The drug susceptibility of *L. donovani* parasites isolated at the end of therapy was comparable to that of pre-treatment isolates. On the contrary, parasites obtained from the cases that relapsed exhibited significantly reduced susceptibility to MIL, although the IC_50_ values were below the expected serum threshold levels [29], implicating the possible involvement of host factors in rendering tolerance to drug. Indeed, reports on VL relapse in HIV co-infected patients treated with MIL suggest that host immunity plays a role in the elimination of parasites from VL patients [30], [31]. A small number of MIL treated cases showed relapse, parasite isolates from these were monitored for *in vitro* drug susceptibility. Although we did not find any clinically resistant strains, the observation of strains with higher MIL tolerance (up to eight times compared to the sensitive ones) emphasizes the need for close monitoring of cases under MIL treatment. The study also investigated for the first time, the intrinsic susceptibility of PKDL isolates towards MIL. The *in vitro* susceptibility of PKDL isolates was significantly higher in pre-treatment isolates than in isolates originating from relapse patients, which were exposed to MIL for long duration (over two months). The IC_50_ of PKDL isolates was significantly higher (∼4 fold) compared to VL isolates, a trend similar to that reported earlier for SAG susceptibility in isolates from high endemic regions [32]. This reduced drug susceptibility of PKDL isolates may be due to longer treatment regime in PKDL and prolonged exposure of parasites to the drug. PMM exhibited similar *in vitro* susceptibility in pre- and post-treatment isolates suggesting its potential in future VL and PKDL therapy.

The inactivation of the genes essential for MIL uptake has been proposed as the simplest mechanism of resistance towards the drug and *L. donovani* MIL transporter LdMT and its subunit LdRos3 have been reported as markers of experimental MIL resistance [28]. Experimentally induced MIL resistant *L.donovani* showed down regulated expression of these transporters [27].

In the current study, a selection of strains from Indian *L. donovani* with variable response to MIL treatment has been tested for four point mutations that were suggested to underlie the development of MIL resistance. No nucleotide exchanges were however, detected in the LdMT and LdRos3 gene fragments sequenced for the set of clinical isolates studied herein, or for clinical isolates from Nepalese MIL relapse cases (data not shown). Screening of whole-genome data revealed that both genes are highly conserved for the examined strains of *L. donovani* from the North of the Indian subcontinent, regardless whether they were isolated from cases responding or not responding to MIL treatment [33]. Comparison of the expression of LdMT and LdRos3 genes revealed a similar expression profile in the different groups of isolates studied herein. Further studies in truly resistant parasites from MIL treated cases, when available, are necessary to explore the possible utility of these genes as markers for monitoring drug susceptibility in clinical isolates. In conclusion, the causative forces leading to MIL resistance cannot be explained by the genomic data available up to date and it is very likely that multi-factorial events may be responsible for the tolerance to chemotherapeutics in *L. donovani* [33].

The study employed an amastigote-macrophage model for monitoring drug susceptibility towards MIL as this stage mimics the host milieu. However, amastigote assays are tedious, time consuming and technically demanding. Hence, drug sensitivity assays based on promastigotes, if found relevant, would be better as simplified biological tool that can be used in clinical settings. The current data recommends for keeping miltefosine susceptibility under close surveillance in the field. The risk of relapse after MIL therapy presses the need for maintenance regimen such as DOT for this oral drug and exploring new drug combinations for regions endemic for VL. Regional policies concerning judicious use of the drug and monitoring the treatment outcome should be implemented and supervised by the health authorities in the endemic areas to minimize the risk of emergence of MIL resistant strains. The development of markers to identify drug unresponsiveness at an early stage constitutes an essential step towards the elimination of this poverty driven disease.

## Supporting Information

Figure S1 **Alignments of sequences for four mutations in the MIL transporter genes potentially linked to** ***in vitro*** **resistance.** The wild-type of the reference strain (MHOM/ET/1967/HU3) is represented in blue color and the reference strain (MHOM/ET/1967/HU3_M) in which miltefosine resistance was experimentally induced in red color. Identified point mutations are labelled in green for verified SNPs previously described. Additional point mutations are marked yellow. Note that BHU902/1 and BHU800/1 could not be amplified with primers designed for locus LdRos3. A) Alignment for locus G630A in the putative *Leishmania donovani* miltefosine transporter. B) Alignment for locus G1261T in the putative *Leishmania donovani* miltefosine transporter. C) Alignment for locus T2567C in the putative *Leishmania donovani* miltefosine transporter. D) Alignment for locus G3T in the putative *Leishmania donovani* miltefosine transporter beta subunit (LdRos3).
(DOC)

Table S1 **Primer sets employed to test for the point mutations of interest.**
(DOCX)
