# Supplementary material for: Drug Susceptibility in Leishmania Isolates Following Miltefosine Treatment in Cases of Visceral Leishmaniasis and Post Kala-Azar Dermal Leishmaniasis
Source: PLoS Negl Trop Dis. 2012 May 22;6(5):e1657. doi: 10.1371/journal.pntd.0001657 (PMC3358331; doi:10.1371/journal.pntd.0001657)
Supplement: Figure S1 — Alignments of sequences for four mutations in the MIL transporter genes potentially linked to in vitro resistance. The wild-type of the reference strain (MHOM/ET/1967/HU3) is represented in blue color and the reference strain (MHOM/ET/1967/HU3_M) in which miltefosine resistance was experimentally induced in red color. Identified point mutations are labelled in green for verified SNPs previously described. Additional point mutations are marked yellow. Note that BHU902/1 and BHU800/1 could not be amplified with primers designed for locus LdRos3. A) Alignment for locus G630A in the putative Leishmania donovani miltefosine transporter. B) Alignment for locus G1261T in the putative Leishmania donovani miltefosine transporter. C) Alignment for locus T2567C in the putative Leishmania donovani miltefosine transporter. D) Alignment for locus G3T in the putative Leishmania donovani miltefosine transporter beta subunit (LdRos3). (DOC) [file pntd.0001657.s001.doc]

Figure S1:

**A)**

NCBI accession number AY321297.1, size 3294bp, chromosome 13, locus tag LdBPK_131590 622408..625701

Mutation: W210*

....|....| ....|....| ....|....| ....|....| ....|....| ....|....| ....|....| ....|

585 595 605 615 625 635 645 655

MHOM/ET/1967/HU3_M TGGACGGCGA GACGAACCTG AAGTCGCGCA AGGCTCTGGA AGCCACCTG**A** GCGCTCTGCG AAGTCGAGGC AATCA

MHOM/ET/1967/HU3 TGGACGGCGA GACGAACCTG AAGTCGCGCA AGGCTCTGGA AGCCACCTG**G** GCGCTCTGCG AAGTCGAGGC AATCA

MHOM/IN/2009/BHU800/1 TGGACGGCGA GACGAACCTG AAGTCGCGCA AGGCTCTGGA AGCCACCTGG GCGCTCTGCG AAGTCGAGGC AATCA

MHOM/IN/2009/BHU994/1 TGGACGGCGA GACGAACCTG AAGTCGCGCA AGGCTCTGGA AGCCACCTGG GCGCTCTGCG AAGTCGAGGC AATCA

MHOM/IN/2010/BHU1093/1 TGGACGGCGA GACGAACCTG AAGTCGCGCA AGGCTCTGGA AGCCACCTGG GCGCTCTGCG AAGTCGAGGC AATCA

MHOM/IN/2009/BHU815/1 TGGACGGCGA GACGAACCTG AAGTCGCGCA AGGCTCTGGA AGCCACCTGG GCGCTCTGCG AAGTCGAGGC AATCA

MHOM/IN/2010/BHU1121/1 TGGACGGCGA GACGAACCTG AAGTCGCGCA AGGCTCTGGA AGCCACCTGG GCGCTCTGCG AAGTCGAGGC AATCA

MHOM/IN/2010/BHU1080/1 TGGACGGCGA GACGAACCTG AAGTCGCGCA AGGCTCTGGA AGCCACCTGG GCGCTCTGCG AAGTCGAGGC AATCA

MHOM/IN/2010/BHU1042/1 TGGACGGCGA GACGAACCTG AAGTCGCGCA AGGCTCTGGA AGCCACCTGG GCGCTCTGCG AAGTCGAGGC AATCA

MHOM/IN/2010/BHU814/1 TGGACGGCGA GACGAACCTG AAGTCGCGCA AGGCTCTGGA AGCCACCTGG GCGCTCTGCG AAGTCGAGGC AATCA

MHOM/IN/2009/BHU741/1 TGGACGGCGA GACGAACCTG AAGTCGCGCA AGGCTCTGGA AGCCACCTGG GCGCTCTGCG AAGTCGAGGC AATCA

MHOM/IN/2010/BHU796/1 TGGACGGCGA GACGAACCTG AAGTCGCGCA AGGCTCTGGA AGCCACCTGG GCGCTCTGCG AAGTCGAGGC AATCA

MHOM/IN/2010/BHU902/1 TGGACGGCGA GACGAACCTG AAGTCGCGCA AGGCTCTGGA AGCCACCTGG GCGCTCTGCG AAGTCGAGGC AATCA

MHOM/IN/2009/BHU1062/4 TGGACGGCGA GACGAACCTG AAGTCGCGCA AGGCTCTGGA AGCCACCTGG GCGCTCTGCG AAGTCGAGGC AATCA

MHOM/IN/2010/BHU1113/7 TGGACGGCGA GACGAACCTG AAGTCGCGCA AGGCTCTGGA AGCCACCTGG GCGCTCTGCG AAGTCGAGGC AATCA

MHOM/IN/2010/BHU872/6 TGGACGGCGA GACGAACCTG AAGTCGCGCA AGGCTCTGGA AGCCACCTGG GCGCTCTGCG AAGTCGAGGC AATCA

**B)**

NCBI accession number AY321297.1, size 3294bp, chromosome 13, locus tag LdBPK_131590 622408..625701

Mutation: T421N

....|....| ....|....| ....|....| ....|....| ....|....| ....|....| ....|....| ....|....| ....|....|

1215 1225 1235 1245 1255 1265 1275 1285 1295

MHOM/ET/1967/HU3_M ACCTCAACGA GCAGCTAGCA ATGGTGCGCT TCATCTTCAG CGACAAAAAT **T**GGACGTTGA CAGAGAACGT CATGAAGTTC AAGCTAGGCG

MHOM/ET/1967/HU3 ACCTCAACGA GCAGCTAGCA ATGGTGCGCT TCATCTTCAG CGACAAAACT **G**GGACGTTGA CAGAGAACGT CATGAAGTTC AAGCTAGGCG

MHOM/IN/2009/BHU800/1 ACCTCAACGA GCAGCTAGCA ATGGTGCGCT TCATCTTCAG CGACAAAACT GGGACGTTGA CAGAGAACGT CATGAAGTTC AAGCTAGGCG

MHOM/IN/2009/BHU994/1 ACCTCAACGA GCAGCTAGCA ATGGTGCGCT TCATCTTCAG CGACAAAACT GGGACGTTGA CAGAGAACGT CATGAAGTTC AAGCTAGGCG

MHOM/IN/2010/BHU1093/1 ACCTCAACGA GCAGCTAGCA ATGGTGCGCT TCATCTTCAG CGACAAAACT GGGACGTTGA CAGAGAACGT CATGAAGTTC AAGCTAGGCG

MHOM/IN/2009/BHU815/1 ACCTCAACGA GCAGCTAGCA ATGGTGCGCT TCATCTTCAG CGACAAAACT GGGACGTTGA CAGAGAACGT CATGAAGTTC AAGCTAGGCG

MHOM/IN/2010/BHU1121/1 ACCTCAACGA GCAGCTAGCA ATGGTGCGCT TCATCTTCAG CGACAAAACT GGGACGTTGA CAGAGAACGT CATGAAGTTC AAGCTAGGCG

MHOM/IN/2010/BHU1080/1 ACCTCAACGA GCAGCTAGCA ATGGTGCGCT TCATCTTCAG CGACAAAACT GGGACGTTGA CAGAGAACGT CATGAAGTTC AAGCTAGGCG

MHOM/IN/2010/BHU1042/1 ACCTCAACGA GCAGCTAGCA ATGGTGCGCT TCATCTTCAG CGACAAAACT GGGACGTTGA CAGAGAACGT CATGAAGTTC AAGCTAGGCG

MHOM/IN/2010/BHU814/1 ACCTCAACGA GCAGCTAGCA ATGGTGCGCT TCATCTTCAG CGACAAAACT GGGACGTTGA CAGAGAACGT CATGAAGTTC AAGCTAGGCG

MHOM/IN/2009/BHU741/1 ACCTCAACGA GCAGCTAGCA ATGGTGCGCT TCATCTTCAG CGACAAAACT GGGACGTTGA CAGAGAACGT CATGAAGTTC AAGCTAGGCG

MHOM/IN/2010/BHU796/1 ACCTCAACGA GCAGCTAGCA ATGGTGCGCT TCATCTTCAG CGACAAAACT GGGACGTTGA CAGAGAACGT CATGAAGTTC AAGCTAGGCG

MHOM/IN/2010/BHU902/1 ACCTCAACGA GCAGCTAGCA ATGGTGCGCT TCATCTTCAG CGACAAAACT GGGACGTTGA CAGAGAACGT CATGAAGTTC AAGCTAGGCG

MHOM/IN/2009/BHU1062/4 ACCTCAACGA GCAGCTAGCA ATGGTGCGCT TCATCTTCAG CGACAAAACT GGGACGTTGA CAGAGAACGT CATGAAGTTC AAGCTAGGCG

MHOM/IN/2010/BHU1113/7 ACCTCAACGA GCAGCTAGCA ATGGTGCGCT TCATCTTCAG CGACAAAACT GGGACGTTGA CAGAGAACGT CATGAAGTTC AAGCTAGGCG

MHOM/IN/2010/BHU872/6 ACCTCAACGA GCAGCTAGCA ATGGTGCGCT TCATCTTCAG CGACAAAACT GGGACGTTGA CAGAGAACGT CATGAAGTTC AAGCTAGGCG

**C)**

NCBI accession number AY321297.1, size 3294bp, chromosome 13, locus tag LdBPK_131590 622408..625701

Mutation: L856P

....|....| ....|....| ....|....| ....|....| ....|....| ....|....| ....|....| ....|....| ....|....|

2525 2535 2545 2555 2565 2575 2585 2595 2600

MHOM/ET/1967/HU3_M TTCAAGCACC TGCGCCGCCT ATGCGCGGTG CATGGCCGCT ACTCGC**C**CTT CCGCAACGCC AGCTGCATTC TGGT**T**AGCTT CCACAAGAAC

MHOM/ET/1967/HU3 TTCAAGCACC TGCGCCGCCT ATGCGCGGTG CATGGCCGCT ACTCGC**T**CTT CCGCAACGCC AGCTGCATTC TGGT**C**AGCTT CCACAAGAAC

MHOM/IN/2009/BHU800/1 TTCAAGCACC TGCGCCGCCT ATGCGCGGTG CATGGCCGCT ACTCGCTCTT CCGCAACGCC AGCTGCATTC TGGTTAGCTT CCACAAGAAC

MHOM/IN/2009/BHU994/1 TTCAAGCACC TGCGCCGCCT ATGCGCGGTG CATGGCCGCT ACTCGCTCTT CCGCAACGCC AGCTGCATTC TGGTTAGCTT CCACAAGAAC

MHOM/IN/2010/BHU1093/1 TTCAAGCACC TGCGCCGCCT ATGCGCGGTG CATGGCCGCT ACTCGCTCTT CCGCAACGCC AGCTGCATTC TGGTTAGCTT CCACAAGAAC

MHOM/IN/2009/BHU815/1 TTCAAGCACC TGCGCCGCCT ATGCGCGGTG CATGGCCGCT ACTCGCTCTT CCGCAACGCC AGCTGCATTC TGGTTAGCTT CCACAAGAAC

MHOM/IN/2010/BHU1121/1 TTCAAGCACC TGCGCCGCCT ATGCGCGGTG CATGGCCGCT ACTCGCTCTT CCGCAACGCC AGCTGCATTC TGGTTAGCTT CCACAAGAAC

MHOM/IN/2010/BHU1080/1 TTCAAGCACC TGCGCCGCCT ATGCGCGGTG CATGGCCGCT ACTCGCTCTT CCGCAACGCC AGCTGCATTC TGGTTAGCTT CCACAAGAAC

MHOM/IN/2010/BHU1042/1 TTCAAGCACC TGCGCCGCCT ATGCGCGGTG CATGGCCGCT ACTCGCTCTT CCGCAACGCC AGCTGCATTC TGGTTAGCTT CCACAAGAAC

MHOM/IN/2010/BHU814/1 TTCAAGCACC TGCGCCGCCT ATGCGCGGTG CATGGCCGCT ACTCGCTCTT CCGCAACGCC AGCTGCATTC TGGTTAGCTT CCACAAGAAC

MHOM/IN/2009/BHU741/1 TTCAAGCACC TGCGCCGCCT ATGCGCGGTG CATGGCCGCT ACTCGCTCTT CCGCAACGCC AGCTGCATTC TGGTTAGCTT CCACAAGAAC

MHOM/IN/2010/BHU796/1 TTCAAGCACC TGCGCCGCCT ATGCGCGGTG CATGGCCGCT ACTCGCTCTT CCGCAACGCC AGCTGCATTC TGGTTAGCTT CCACAAGAAC

MHOM/IN/2010/BHU902/1 TTCAAGCACC TGCGCCGCCT ATGCGCGGTG CATGGCCGCT ACTCGCTCTT CCGCAACGCC AGCTGCATTC TGGTTAGCTT CCACAAGAAC

MHOM/IN/2009/BHU1062/4 TTCAAGCACC TGCGCCGCCT ATGCGCGGTG CATGGCCGCT ACTCGCTCTT CCGCAACGCC AGCTGCATTC TGGTTAGCTT CCACAAGAAC

MHOM/IN/2010/BHU1113/7 TTCAAGCACC TGCGCCGCCT ATGCGCGGTG CATGGCCGCT ACTCGCTCTT CCGCAACGCC AGCTGCATTC TGGTTAGCTT CCACAAGAAC

MHOM/IN/2010/BHU872/6 TTCAAGCACC TGCGCCGCCT ATGCGCGGTG CATGGCCGCT ACTCGCTCTT CCGCAACGCC AGCTGCATTC TGGTTAGCTT CCACAAGAAC

**D)**

NCBI accession number DQ205096.1, size 1098bp, chromosome 32, locus tag LDBPK_320540 186186.187277

Mutation: M1*

....|....| ....|....| ....|....| ....|....| ....|....| ....|....| ....|....| ....|....| ....|....|

-35 -25 -15 -5 5 15 25 35 45

MHOM/ET/1967/HU3_M TTCTTTCTTT TTTCTGCTGC TGTTACTCGA GGGCCAAATC AT**T**GCGCCTC TACCCCCTAA GCCACACTCG AAAAACCGCA TTGAGCAGCA

MHOM/ET/1967/HU3 TTCTTTCTTT TTTCTGCTGC TGTTACTCGA GGGCCAAATC AT**G**GCGCCTC TACCCCCTAA GCCACACTCG AAAAACCGCA TTGAGCAGCA

MHOM/IN/2009/BHU994/1 TTCTTTCTTT TTTCTGCTGC TGTTACTCGA GGGCCAAATC ATGGCGCCTC TACCCCCTAA GCCACACTCG AAAAACCGCA TTGAGCAGCA

MHOM/IN/2010/BHU1093/1 TTCTTTCTTT TTTCTGCTGC TGTTACTCGA GGGCCAAATC ATGGCGCCTC TACCCCCTAA GCCACACTCG AAAAACCGCA TTGAGCAGCA

MHOM/IN/2009/BHU815/1 TTCTTTCTTT TTTCTGCTGC TGTTACTCGA GGGCCAAATC ATGGCGCCTC TACCCCCTAA GCCACACTCG AAAAACCGCA TTGAGCAGCA

MHOM/IN/2010/BHU1121/1 TTCTTTCTTT TTTCTGCTGC TGTTACTCGA GGGCCAAATC ATGGCGCCTC TACCCCCTAA GCCACACTCG AAAAACCGCA TTGAGCAGCA

MHOM/IN/2010/BHU1080/1 TTCTTTCTTT TTTCTGCTGC TGTTACTCGA GGGCCAAATC ATGGCGCCTC TACCCCCTAA GCCACACTCG AAAAACCGCA TTGAGCAGCA

MHOM/IN/2010/BHU1042/1 TTCTTTCTTT TTTCTGCTGC TGTTACTCGA GGGCCAAATC ATGGCGCCTC TACCCCCTAA GCCACACTCG AAAAACCGCA TTGAGCAGCA

MHOM/IN/2010/BHU814/1 TTCTTTCTTT TTTCTGCTGC TGTTACTCGA GGGCCAAATC ATGGCGCCTC TACCCCCTAA GCCACACTCG AAAAACCGCA TTGAGCAGCA

MHOM/IN/2009/BHU741/1 TTCTTTCTTT TTTCTGCTGC TGTTACTCGA GGGCCAAATC ATGGCGCCTC TACCCCCTAA GCCACACTCG AAAAACCGCA TTGAGCAGCA

MHOM/IN/2010/BHU796/1 TTCTTTCTTT TTTCTGCTGC TGTTACTCGA GGGCCAAATC ATGGCGCCTC TACCCCCTAA GCCACACTCG AAAAACCGCA TTGAGCAGCA

MHOM/IN/2010/BHU902/1 TTCTTTCTTT TTTCTGCTGC TGTTACTCGA GGGCCAAATC ATGGCGCCTC TACCCCCTAA GCCACACTCG AAAAACCGCA TTGAGCAGCA

MHOM/IN/2010/BHU1113/7 TTCTTTCTTT TTTCTGCTGC TGTTACTCGA GGGCCAAATC ATGGCGCCTC TACCCCCTAA GCCACACTCG AAAAACCGCA TTGAGCAGCA

MHOM/IN/2010/BHU872/6 TTCTTTCTTT TTTCTGCTGC TGTTACTCGA GGGCCAAATC ATGGCGCCTC TACCCCCTAA GCCACACTCG AAAAACCGCA TTGAGCAGCA
