# Supplementary material for: Drug Susceptibility in Leishmania Isolates Following Miltefosine Treatment in Cases of Visceral Leishmaniasis and Post Kala-Azar Dermal Leishmaniasis
Source: PLoS Negl Trop Dis. 2012 May 22;6(5):e1657. doi: 10.1371/journal.pntd.0001657 (PMC3358331; doi:10.1371/journal.pntd.0001657)
Supplement: Table S1 — Primer sets employed to test for the point mutations of interest. (DOCX) [file pntd.0001657.s002.docx]

Table S1: **Primer sets employed to test for the point mutations of interest.**

| Amino acid ^1^ | Nt position ^2^ | Chr ^3^ | Primer sequences ^5'^------------------------^3'^ | T_A_ (°C) ^4^ | Amplicon size (bp) | Accession number ^5^ | Gene ID ^6^ | Mutational effect |
| --- | --- | --- | --- | --- | --- | --- | --- | --- |
| W210* | G630A | 13 | fwd: CGAGGAAGGACAGGCATTTA  rev: GGTCTGGCTTGCTCGTGTC | 56 | 149 | AY321297.1 | LdBPK_ 131590 | affects protein folding and/or |
| T421N | G1261T | 13 | fwd: CTGCCTCATGATGGAGTACA  rev: GCCTAGCCCCTTCGACTC | 58 | 235 | AY321297.1 | LdBPK_ 131590 | expression stop  truncated protein |
| L856P | T2567C | 13 | fwd: CCAACGACGTGTCCATGAT  rev: AAGGTCAGCATCCATCCATC | 58 | 277 | AY321297.1 | LdBPK_ 131590 | recognition of LdMT |
| M1* | G3T | 32 | fwd: TACAGCTTTTGCTGCCCTTT  rev: ATAGCAGCGACTGCCAGAAT | 58 | 236 | DQ205096.1 | LdBPK_320540 | start codon mutation  truncated protein |

The presence of two nonsense and two missense mutations was tested in a representative set of *L. donovani* isolates from Bihar, India. ^1^ amino acid position, ^2^ nucleotide position indicating the 'natural' nucleotide and the point mutation, ^3^ chromosome, ^4^ annealing temperature, Note: ^5^ Accession number corresponds to the published sequences of the LdMT and LdRos3 genes obtained for the *L. donovani* strain MHOM/ET/1967/HU3 (Perez-Victoria 2006), ^6^ Gene ID corresponds to that for the *L. donovani* complete reference genome strain BPK282A1.
